# Supplementary material for: Early observations from the HIV self-testing program among key populations and sexual partners of pregnant mothers in Kampala, Uganda: A cross sectional study
Source: PLOS Glob Public Health. 2022 Jan 6;2(1):e0000120. doi: 10.1371/journal.pgph.0000120 (PMC10021783; doi:10.1371/journal.pgph.0000120)
Supplement: Attachment — Submitted filename: Response to Reviewers and Editor 01 11 2021.docx [file pgph.0000120.s003.docx]

**Response to Editor’s comments**

Journal Requirements:

| No | Comment | Response |
| --- | --- | --- |
| 1. | Please provide separate figure files in .tif or .eps format only, and remove any figures embedded in your manuscript file. | Separate files have been provided.  There are no figures embedded in the manuscript. |
| 2. | Please update the completed 'Competing Interests' statement, including any COIs declared by your co-authors. If you have no competing interests to declare, please state "The authors have declared that no competing interests exist". Otherwise please declare all competing interests beginning with the statement "I have read the journal's policy and the authors of this manuscript have the following competing interests:" | This has been changed in the text of the manuscript to show that “The authors have declared that no competing interests exist”  See lines 216 and 217 |
| 3 | In the online submission form, you indicated that "Data is available from the corresponding author upon request."  All PLOS journals now require all data underlying the findings described in their manuscript to be freely available to other researchers, either 1. In a public repository, 2. Within the manuscript itself, or 3. Uploaded as supplementary information. This policy applies to all data except where public deposition would breach compliance with the protocol approved by your research ethics board. If your data cannot be made publicly available for ethical or legal reasons (e.g., public availability would compromise patient privacy), please explain your reasons by return email and your exemption request will be escalated to the editor for approval. Your exemption request will be handled independently and will not hold up the peer review process, but will need to be resolved should your manuscript be accepted for publication. One of the Editorial team will then be in touch if there are any issues. | The data has been uploaded as supplementary information. |
| 4. | Please amend your detailed Financial Disclosure statement. This is published with the article, therefore should be completed in full sentences and contain the exact wording you wish to be published.  If you did not receive any funding for this study, please simply state: “The authors received no specific funding for this work.” | This has been amended to read “The authors received no specific funding for this work”. See lines 218 and 219 |
|  | Additional Editor Comments (if provided) |  |
| 5. | STROBE guidelines should be adhered to, e.g. describe the design in title and abstract. Please complete the STROBE checklist for cross-sectional studies and submit with a revised version along with a flow chart of patients enrolled | The STROBE checklist has been added and a flow chart included in the manuscript files. |
| 6 | While revising your submission, please upload your figure files to the Preflight Analysis and Conversion Engine (PACE) digital diagnostic tool, <https://pacev2.apexcovantage.com/>. PACE helps ensure that figures meet PLOS requirements. To use PACE, you must first register as a user. Registration is free. Then, login and navigate to the UPLOAD tab, where you will find detailed instructions on how to use the tool. If you encounter any issues or have any questions when using PACE, please email PLOS at [figures@plos.org](mailto:figures@plos.org). Please note that Supporting Information files do not need this step. | The two figures have been uploaded to PACE. |

Response to Review Comments to the Author

Reviewer #1:

|  | Comment | Response |
| --- | --- | --- |
| 1 | Introduction Shouldn’t “first 95” be defined more clearly p. 3 line 30: “they continue to lag behind other populations in the first 95 in Eastern….”? | The paragraph has been rearranged to first define the first 95 so that it has better context when mentioned in regards to the key populations later.  See line 27 to 30. |
| 2 | Methods What are “emancipated minors (16-<18 years)”? | Emancipated minors are those who have children or are heads of households. This has been made explicit in the manuscript text. See line 70 and 71 |
| 3 | p. 5 l. 69 “peer-to-peer model for KPs” – doesn’t this introduce a bias? From social network studies it looks like information spreads to people who like you and are part of the same mindsets? | Due to legal and structural barriers for KPs in Uganda, the KP population is a hidden one. They are very hard to access for service provision. They also have a lot of mistrust for almost everyone. Using their peers to approach them and provide services has helped with the access and mistrust issues and led to program success. |
| 4 | p. 5 l. 72 “to their partners with unknown HIV status at home…” – how do they know it is unknown? Could this be a cause for overestimation of effect? -Is there a possible bias in the fact that knowing who the father (?) or partner is/having a partner could be a social determinant for health, empowerment or health literacy? | The status is unknown to the mothers. At this point, we do not know if the partners genuinely don’t know the status.  We do not think that the effect is overestimated especially since we do not report any measures of association. |
| 5 | p.5 l. 81. How was partner violence handled in terms of home testing? – is it likely that a violent partner would accept home testing by partner? | All mothers were screened for intimate partner violence and those with a history of partner violence were screened further by a trained counsellor to determine who could be supported to go through the HIVST process with a health worker or to halt the exercise all together. This has been made clearer in the manuscript. See line 86 to 88 |
| 6 | p. 5 l. 84 “KP peers trained in HIVST, distributed HIVST kits in the community to KPs” – what does this mean exactly? | KP peers were trained prior to being given the kits. See excerpt from the manuscript draft.  *Selected health workers, including midwives, and peer distributors at participating health facilities received 3-day trainings prior to distribution of HIVST kits. The training focused on self-test kit use, importance of HIV positive result confirmation, management of tests including storage and documentation.*  Lines 77 to 80 |
| 7 | How were KPs with unknown HIV status identified? | The KPS were screened using the standard Uganda National Adult HIV Testing Services screening tool. This tool determines eligibility for an HIV test based on particular risk factors and duration since the last test. Those who screened positive (unknown HIV status or > 3 months since the last test for KPS) were given test kits. We have made it clearer in the manuscript.  Lines 91 to 94 |
| 8 | How were FSW and MSM identified and how likely are they to openly identify as such in Uganda? Were there no fears of being registered for other purposes? | The KP peers identified and distributed kits to peers. The KP peers are members of the KP community in Uganda, that is they are either MSM or FSWs themselves and that is why they were chosen. They know and live with fellow peers and they trust them. This way, the fellow KPs disclose to them with no fear.  We have made this clearer in the manuscript. Lines 74 and 75 |
| 9 | p. 5 l. 87 “KP peers trained in HIVST, distributed HIVST kits in the community to KPs” – could there be other ‘convenient meeting points for KPs? Not clear to me if partners are also included in MSM/FSW ? | The KPs are better targeted in the community as at the facility they do not identify as such.  No, partners of the MSM and FSWs are not included. |
| 10 | Results p. 6 l. 109 These figures are not clear to me: “Most (6965 [74%]) to women aged 110 16 to 24 years (4496 [48%])” – what is 6965 and what is 4496?) . it is clearer in the table so the text is too condensed. | A flow chart has been added that explains these figures better.  See figure 1 |
| 11 | p. 6 l. 110 “Kits used by male sexual partners of mothers were 5212 (56%)” – but % of which number? | This was as a fraction of all the HIVST that were distributed. (9,378). This is also clearer in the flow chart. See figure 1 |
| 12 | Discussion What is meant by “early observations”? – please clarify | Early observations as the program is just starting and was less than one year old at the time that the data was analysed. It could actually change as the program matures and if we did this analysis now, two years later, we might see different results. |
| 13 | Would partners/MSM/FSW that know (or suspect) to be infected be more likely not to participate? | We can only speculate on this and do not have a definite answer. |
| 14 | Some of the shortcomings in methods and results are caused by the fact that the study is based on routine data that were collected for general purposes. What would have been done differently if the study had been designed to collect the relevant data? | We could have collected data on the partners of the FSWs and MSMs. We could have found out the reasons why people who already know their HIV status are trying to pass it off as unknown and assessed the optimal methods for linkage. We could also have collected the data on those who refused to take the HIVSTs to their partners or for their own test (KP). |

Reviewer #2: Nasuuna et al

| No | Comment | Response |
| --- | --- | --- |
| 1 | This paper provides findings from an HIV self-testing (HIVST) programme focussed on key populations and sexual partners of pregnant mothers in Kampala, Uganda, reporting HIV prevalence by group and subsequent rates of linkage to HIV treatment for those testing HIV positive. This is an important research area, and the paper is generally well written. However I do have a number of points which hopefully would improve the analysis and its presentation, particularly regarding full presentation of all the relevant data. | Thank you very much for your review and the suggestions on how to make this better.  Hopefully, we have addressed them sufficiently to make the paper better. |
|  | Major comments |  |
| 2 | Throughout the manuscript, percentage linked to care is presented using the denominator of PLHIV confirmed HIV positive. I think authors should present percentage linked to care using the denominator of all those testing positive using HIVST, so there should be two linkage percentages reported for each group: percentage with HIVST-positive results whose HIV positivity is confirmed and percentage of those linked to care. | The data is reported this way in keeping with the care cascade that is followed in Uganda. Additionally, we can only speculate as to the true HIV status and linkage status of those who did not report for confirmation or linkage. It is possible that they were linked to other health centres that we do not have access to and it would be erroneous to count them on this side. They could also have wrongly reported their results. Therefore, we considered it better to report for the ones we are very sure about. However, we have mentioned a new percentage of 50% if we assume that all who reported HIV positive results were truly positive. This has been added to the manuscript. See line 138 to 139 |
| 3 | Only half (126/252) of HIVST-positive individuals linked to care. This deserves more prominence. The Discussion (p9, line 155) states that linkage is higher than reported in Malawi (56%, ref 23) and South Africa (67%, ref 14). I have quickly checked ref 23, and the denominator for this seems to be all those testing positive by HIVST – so actually higher than the linkage reported here. I have not checked ref 14, but I strongly advise the authors to do so and check that they are comparing like with like | We have put more information in pertaining to these two studies in the manuscript.  We have elaborated on exactly what we are comparing. Lines 52 to 55 |
| 4 | The reason for the drop from 252 HIV-positives by HIVST to 170 confirmed HIV-positive should be made much clearer – how many of the 252 returned for confirmatory testing and how many were treated as HIVST false positives? The diagnostic accuracy of HIVST is also not stated (sensitivity, specificity) and its implications not discussed. | The sensitivity and specificity of the OraQuick HIVST has been added as found in a prior Ugandan study. Lines 55 to 56  The rest of the information is not available as this was use of routinely collected data which did not capture that information. [Nangendo, 2017 #36] |
| 5 | What percentage didn’t bring back the OraQuick paddle? What percentage didn’t present for confirmatory testing and were tested through a community visit? | This information is not available from the routine medical records. |
| 6 | Methods p5 line 70: how were the peers recruited? | The facilities had other existing KP interventions such as PrEP, increasing accessing of KPs to HIV health services through peer mobilizers and linkage facilitators that were working closely with the KP community using the KP peers. The peers running these programs were utilized to also conduct HIVST. Since the peers are members of the KP community, snowballing was used to reach the necessary number at the start of the overall KP program. |
| 7 | Only those of unknown HIV status were offered HIVST. This deserves discussion. HIV status is dynamic, and it may be known to a mother’s sexual partner, but not necessarily to the mother. 17 of those reporting their HIV status already knew they were HIV positive but had not disclosed it to their partners/KP peers – surely even more individuals who report being HIV negative are actually not disclosing their status, or are unaware of their status? | This has been mentioned under limitations of the study. We need better ways of collecting the HIV status information. Lines 187 to 189 |
| 8 | Methods p5 line 88: What number of KPs refused to provide a telephone number, and if so, were they just excluded from the analysis or were they also refused a kit? | Unfortunately, this information is not reported in the routine data collection tools that were used for this analysis. |
| 9 | Results: I would recommend a flow chart figure to summarise the numbers of peer and mother distributors, numbers of kits distributed and number of recipients, stratified by KP type (FSW, MSM) and sexual partner. The flowchart could then show numbers and % with HIVST-positive results, numbers/% for confirmatory testing and finally, numbers/% starting HIV treatment. It would make the outcomes much clearer. | A flow chart has been added to make the outcomes clearer. See figure 2 |
| 10 | The table could also be made more informative by adding extra columns – the first for all, then further columns for FSW, MSM and sexual partners of mothers. | We are limited by the lack of individual level data as most program data is reported as aggregated data. This might not be possible. |
| 11 | It seems that 100% of the 9378 HIVST kits are accounted for, with recipients reporting their results. Surely there were some kits distributed and lost. Please report these in the text and/or flowchart. | All effort was made to ensure that all the kits distributed were accounted for. That is why we had 100% reporting of the kits distributed. No kits were reported lost. |
| 12 | I think the Discussion needs to go into more detail on why people are lost at each step in the pathway and the limitations of the analysis (e.g. only including those with unknown HIV status). | Unfortunately, one of the limitations of the current national data capture tools is that they do not allow for the collection of data on people that refuse to join the program, those that join and then leave and the reasons for this. We have highlighted this as a limitation of the current program in the discussion that needs to be addressed. See lines 187 to 191  The HIVST program in Uganda is meant to decrease the number of key and priority populations that do not know their HIV status. Therefore, we do not think that it is a limitation to include only those with unknown HIV status. They are the target of the program. |
|  | Once there is more transparent reporting on numbers excluded, number of kits distributed per KP distributor etc, the Discussion should then make some fuller explanations for why linkage is so poor and suggest next steps to try to improve it. I think using large parts of the Discussion to compare HIV positivity rates with other countries’ (p9, first paragraph) is of less utility. | This information is not available in the routine national data tools that were used for this analysis. This has already been highlighted as a limitation of the current study.  We have suggested ways to improve linkage in the manuscript. See lines 177 to 179 |
| 13 | The figure could be presented in a more compelling way. In its current form, the 79% and 99 figures overlap and it’s unclear (changing the scale of the right hand y axis would solve that). However I think it would look better as a stacked bar chart, one bar for each population (FSW, MSM and partners) and either all the same height (so y axis is percentages, and numbers of PLHIV could be written on the chart), or different heights (y axis is number of PLHIV and percentages are written on the chart). | The figure has been redrawn into a stacked bar graph. We hope that it is clearer now.  See figure 2 |
|  | Minor comments |  |
| 14 | Introduction line 3: “in the first 95” sounds a little unclear here, out of context – it’s only explained later, on p33. | This has been clarified and reworded in the introduction to make it clearer. See lines 27 to 30 |
| 15 | Last paragraph of the Introduction: authors cite refs 18 and 19 reporting HIVST for KP and mothers’ partners in Uganda. They should use this point in the paper to explain what insights their analysis provides above and beyond these two papers. | It has been made clear that these were RCTs with very controlled conditions and our study reports implementation in a real-world setting. Lines 52 to 53 |
| 16 | Methods p5 line 82: mothers at risk of IPV were “supported accordingly”. Please give brief details of this support and state whether these mothers were then excluded. | Screening to determine IPV risk using a GBV screening tool, determine whether to give individual a kit to take to partner or not, those who need for support against IPV are referred for more appropriated services.  Kits are given to only those where its determined that there is no risk.  This has been clarified in the manuscript. See lines 86 to 88 |
| 17 | Methods p5 line 86: What are KP dens, and would these be different for FSW and MSM populations? | KP hot spots- community spaces where the FSWs and MSMs hang out, stay or where they often congregate. They could be different for the different KP categories but the peers would know where to find them. |
| 18 | Methods p6 line 91: “mothers and peers were asked to confirm delivery” – how? Telephone? | Yes, through the telephone. This has been clarified in the manuscript. Lines 101 to 102 |
| 19 | Methods p6 line 94: Probably could delete “immediately”, since later in the sentence the time window is reported as “within two days”. | The word immediately has been deleted. Line 104 |
| 20 | Discussion, 2nd sentence: “facility-based distribution was more utilized…” I think we need to see more data to confirm this. Firstly, make it clearer in the table that facility-based distribution was for mothers, and community-based distribution was for KPs | This has been made clearer in the table.  We also think there was more utilisation of the facility because the mothers were more than the KPs. |
| 21 | I would also want to know how many mothers and KPs were offered but declined tests (and what proportion were deemed ineligible for having no partners/peers, or only partners/peers of known HIV status). | Unfortunately, the data capture tools (kit distribution log) only capture kits distributed and not kits declined. |
| 22 | Also, how many distributors were there in each group? | The number of distributers keeps changing as there are always different numbers of women attending the ante natal clinic. The KP peers work on a rotational basis with one peer serving more than one KP hot spot. |
| 23 | Discussion p9 line 148: Authors state that Malawi has a higher HIV prevalence than Uganda at 10.6%. State the prevalence in Uganda here as well. The discussions in this paragraph of positivity rates: worth quoting the uncertainty bounds. | The prevalence in Uganda has been added. Line 164  The confidence bands for the positivity rate have been added in the result section. Line 127 |
| 24 | Discussion: I’d recommend cost-effectiveness analysis as a future research direction. | Thank you for this suggestion. We have added this to our recommendations in the conclusion. Line 198 to 199 |
| 25 | Discussion last sentence: Rephrase “policy for improvement”. | This has been rephrased to say national policy changes to improve linkage to care. Line 201 |
