# Supplementary material for: Early observations from the HIV self-testing program among key populations and sexual partners of pregnant mothers in Kampala, Uganda: A cross sectional study
Source: PLOS Glob Public Health. 2022 Jan 6;2(1):e0000120. doi: 10.1371/journal.pgph.0000120 (PMC10021783; doi:10.1371/journal.pgph.0000120)
Supplement: Attachment — Submitted filename: Response to Reviewers 18 11 2021.docx [file pgph.0000120.s004.docx]

Response to Reviewers

Journal Requirements:

Please review your reference list to ensure that it is complete and correct. If you have cited papers that have been retracted, please include the rationale for doing so in the manuscript text, or remove these references and replace them with relevant current references. Any changes to the reference list should be mentioned in the rebuttal letter that accompanies your revised manuscript. If you need to cite a retracted article, indicate the article’s retracted status in the References list and also include a citation and full reference for the retraction notice.

Response: I have run a reference check through Scite which has showed that none of the references were retracted or have any concern.

Additional Editor Comments (if provided):

The STROBE checklist only states that the items are completed, but it should be added where in the manuscript, so it is possible to evaluate if the item is properly dealt with

Response: The checklist has been updated to show exactly where all the items were addressed. See attached STROBE list.

Reviewers' comments:

Response: There were no additional reviewer comments.
